# Perioperative Interventions for Prevention of Prolonged Postoperative Ileus and Gastrointestinal Recovery After Colorectal Surgery: A Systematic Review of Randomized Controlled Trials

**DOI:** 10.3390/jcm15218532

**Published:** 2026-11-02

**Authors:** Cristian-Ștefan Hleșcu, Ioana Creangă-Murariu, Andreea Laura Antohi, Andreea-Alexandra Hleșcu, Ana Simona Barna, Cristina Maria Muzică, Roxana Grigorovici, Alina Călin Frij, Bogdan-Ionel Tamba, Alexandru Grigorovici

**Affiliations:** 1 Department of Surgery, Faculty of Medicine, Grigore T. Popa University of Medicine and Pharmacy, 16 University Street, 700115 Iasi, Romania; cristian.stefan.hlescu@umfiasi.ro (C.-Ș.H.); alina.frij-calin@umfiasi.ro (A.C.F.); ale.grigorovici@umfiasi.ro (A.G.); 2 Advanced Research and Development Center for Experimental Medicine “Prof. Ostin C. Mungiu” (CEMEX), Grigore T. Popa University of Medicine and Pharmacy, 16 University Street, 700115 Iasi, Romania; antohi.andreea31@yahoo.com (A.L.A.); bogdan.tamba@umfiasi.ro (B.-I.T.); 3 Medical Oncology-Radiotherapy Department, Grigore T. Popa University of Medicine and Pharmacy, 16 University Street, 700115 Iasi, Romania; 4 Centre for Translational Medicine, Semmelweis University, 20 Baross Street, 1085 Budapest, Hungary; grigorovici.roxana@d.umfiasi.ro; 5 Department of Legal Medicine, Faculty of Medicine, Grigore T. Popa University of Medicine and Pharmacy, 16 University Street, 700115 Iasi, Romania; 6 Faculty of Chemical Engineering and Environmental Protection “Cristofor Simionescu”, “Gheorghe Asachi” Technical University, 71A Mangeron Blvd., 700050 Iasi, Romania; ana-simona.barna@academic.tuiasi.ro; 7 Department of Gastroenterology, Faculty of Medicine, Grigore T. Popa University of Medicine and Pharmacy, 16 University Street, 700115 Iasi, Romania; cristina-maria.muzica@umfiasi.ro; 8 Department Gastroenterology, St. Spiridon Hospital, 700111 Iasi, Romania; 9 Department of Surgery I, St. Spiridon Hospital, 700111 Iasi, Romania

**Keywords:** prolonged postoperative ileus, perioperative interventions, colorectal surgery, early predictors

## Abstract

**Background:** Prolonged postoperative ileus (PPOI) remains a common complication after colorectal surgery, leading to delayed gastrointestinal recovery, prolonged hospitalization, and increased healthcare costs. Despite minimally invasive surgery and enhanced recovery protocols, postoperative gastrointestinal dysfunction remains frequent. Interpretation of current evidence is limited by substantial heterogeneity in PPOI definitions and outcome reporting. **Methods:** This systematic review was conducted according to PRISMA 2020 and Cochrane Handbook recommendations, following a protocol prospectively registered for PROSPERO (CRD420261307668). PubMed/MEDLINE, Embase, and CENTRAL were searched for randomized controlled trials published since January 2000 involving adult colorectal surgery patients. Studies evaluating all perioperative factors associated with PPOI were included. Screening, data extraction, and risk-of-bias assessment were independently performed by multiple reviewers. Findings were synthesized narratively according to perioperative phase and intervention type. **Results:** Of the 36 included RCTs, 7 (19.4%) reported an operational definition of POI/PPOI, of which 5 (13.9% of all included trials) applied a predefined time-based definition corresponding specifically to PPOI. The remaining studies reported POI/PPOI without sufficiently detailed diagnostic criteria or primarily assessed surrogate measures of gastrointestinal recovery. Across the included trials, effects on postoperative gastrointestinal recovery were heterogeneous, although selected analgesia strategies, early oral feeding, gum chewing, and selected pharmacological interventions showed potentially favorable effects on gastrointestinal recovery outcomes. Non-opioid and multimodal analgesic strategies were associated with earlier bowel recovery, with time to first flatus within 1.5–2 days and first bowel movement within 2–3 days. Although leukocyte count and CRP remained elevated postoperatively in the limited number of trials reporting these markers, no significant between-group differences were observed; therefore, these data do not establish an independent association between postoperative inflammation and PPOI. **Conclusions:** Because clinically defined PPOI was assessed in only a small proportion of included trials, the available evidence relates predominantly to postoperative gastrointestinal recovery rather than to prevention of clinically defined PPOI. PPOI remains a multifactorial complication arising within the complex interplay of surgical, pharmacological, inflammatory, and perioperative factors; however, the independent effects of opioid exposure, postoperative inflammation, and background recovery protocols cannot be established from the included trials unless these factors are directly randomized. Potential signals of benefit were observed with selected analgesic strategies, early feeding, gum chewing, and some pharmacological interventions; however, heterogeneity in PPOI definitions, interventions, and outcome reporting precludes definitive conclusions regarding their efficacy for PPOI prevention. Persistent heterogeneity in PPOI definitions limits direct comparison between studies. The randomized intervention–control comparisons identified potential signals of benefit for selected strategies, although their efficacy for preventing clinically defined PPOI remains uncertain.

## 1. Introduction

Prolonged postoperative ileus (PPOI) remains a major impediment to recovery after colorectal surgery [1,2]. Although often treated as a routine postoperative inconvenience, PPOI is better understood as a clinically consequential failure of gastrointestinal recovery, associated with intolerance of oral intake, delayed recovery, prolonged hospitalization and increased healthcare resource utilization [1,3]. The condition is further complicated by persistent inconsistency in the distinction between expected postoperative gut dysmotility and pathological ileus [4]. This distinction is not trivial. In colorectal surgery, where recovery pathways are explicitly designed to accelerate restoration of gut function [5], postoperative ileus (POI) continues to occur with substantial frequency. Reported incidence after colon and rectal surgery commonly ranges from 10% to 30% for POI [2,6,7] and approximately 10% for PPOI [8]. The continued prominence of ileus within perioperative care pathways is itself revealing: despite minimally invasive surgery and multimodal recovery protocols designed to hasten bowel recovery, postoperative gastrointestinal dysfunction remains a persistent clinical problem [2,5,9].

One reason durable progress has been difficult is that PPOI has never been studied against a truly stable clinical construct. Across the literature, the terminology and operational definitions used to describe POI are inconsistent, while the outcomes chosen to represent it vary markedly, including time to first flatus, time to first bowel movement, tolerance of diet, nasogastric tube reinsertion, abdominal distension, radiologic confirmation, prolonged length of stay and composite institutional definitions [1,4,10]. Although a clinically anchored day-4 definition was proposed more than a decade ago [4], PPOI remains inconsistently defined today [1]. The literature remains weighted toward surrogate recovery endpoints rather than direct, standardized definitions of postoperative ileus [10].

This methodological inconsistency is especially problematic because PPOI is not a unitary phenomenon but the clinical expression of a layered perioperative pathobiology. Contemporary mechanistic work supports at least two interlocking phases: an early neurogenic phase, driven by inhibitory neural reflexes initiated by surgical trauma, and a later inflammatory phase, sustained by bowel manipulation, activation of resident muscularis macrophages, leukocyte recruitment and neuroimmune crosstalk within the intestinal wall [11]. Resident macrophages, enteric glia and enteric neurons have emerged as central effectors in this cascade, while mast cells have also been implicated [12,13]. Cytokine signaling and enteric gliosis are now recognized as active contributors rather than epiphenomena [14,15].

That mechanistic architecture gives coherence to the wide spectrum of perioperative variables repeatedly implicated in colorectal PPOI. Recent syntheses identify older age, male sex, previous abdominal surgery, ileostomy creation, open surgery and prolonged operative time among the most reproducible risk factors for POI after colorectal surgery [16,17]. Cohort-level reports additionally implicate greater blood loss or transfusion requirement, liberal perioperative fluid administration, more extensive surgery, postoperative opioid exposure and failure of early mobilization [9,18,19]. Importantly, these are not isolated predictors as they map onto distinct biological pressures acting before, during and after surgery [11,20]. Preoperative status may influence baseline vulnerability [21]. Intraoperative factors shape the magnitude of tissue trauma, bowel manipulation and physiological stress [19]. Postoperative care determines the degree to which neural inhibition, immobility, opioid-related dysmotility and delayed enteral stimulation are allowed to persist [9,22]. A large number of strategies have been proposed to prevent or attenuate PPOI, but nevertheless, the syndrome persists because interventions are being tested against endpoints that are mechanistically related but not nosologically equivalent. In other words, the problem is not simply that PPOI is difficult to treat but that it is still imperfectly defined and variably operationalized and is, therefore, inconsistently captured across trials.

Accordingly, any meaningful synthesis of the literature must do more than catalogue whether an intervention shortens time to flatus or hospital stay. This review aimed to determine whether perioperative interventions reduce the incidence of PPOI and improve gastrointestinal recovery after colorectal surgery. It must also ask whether the measured endpoint reflects true prevention of PPOI, accelerated physiological recovery or merely a proxy for one component of postoperative bowel function. The rationale for the present review arises precisely from this gap. By examining preoperative, intraoperative and postoperative factors in relation to the way PPOI was defined or, in many cases, not explicitly defined, we sought to interpret the literature not as a collection of disconnected perioperative tactics but as evidence generated around a biologically coherent yet methodologically unstable postoperative syndrome. Such an approach is necessary if future colorectal studies are to move beyond descriptive recovery markers toward a standardized, clinically meaningful and mechanistically grounded understanding of prolonged postoperative ileus.

## 2. Materials and Methods

### 2.1. Data Sources

**Figure 1:**
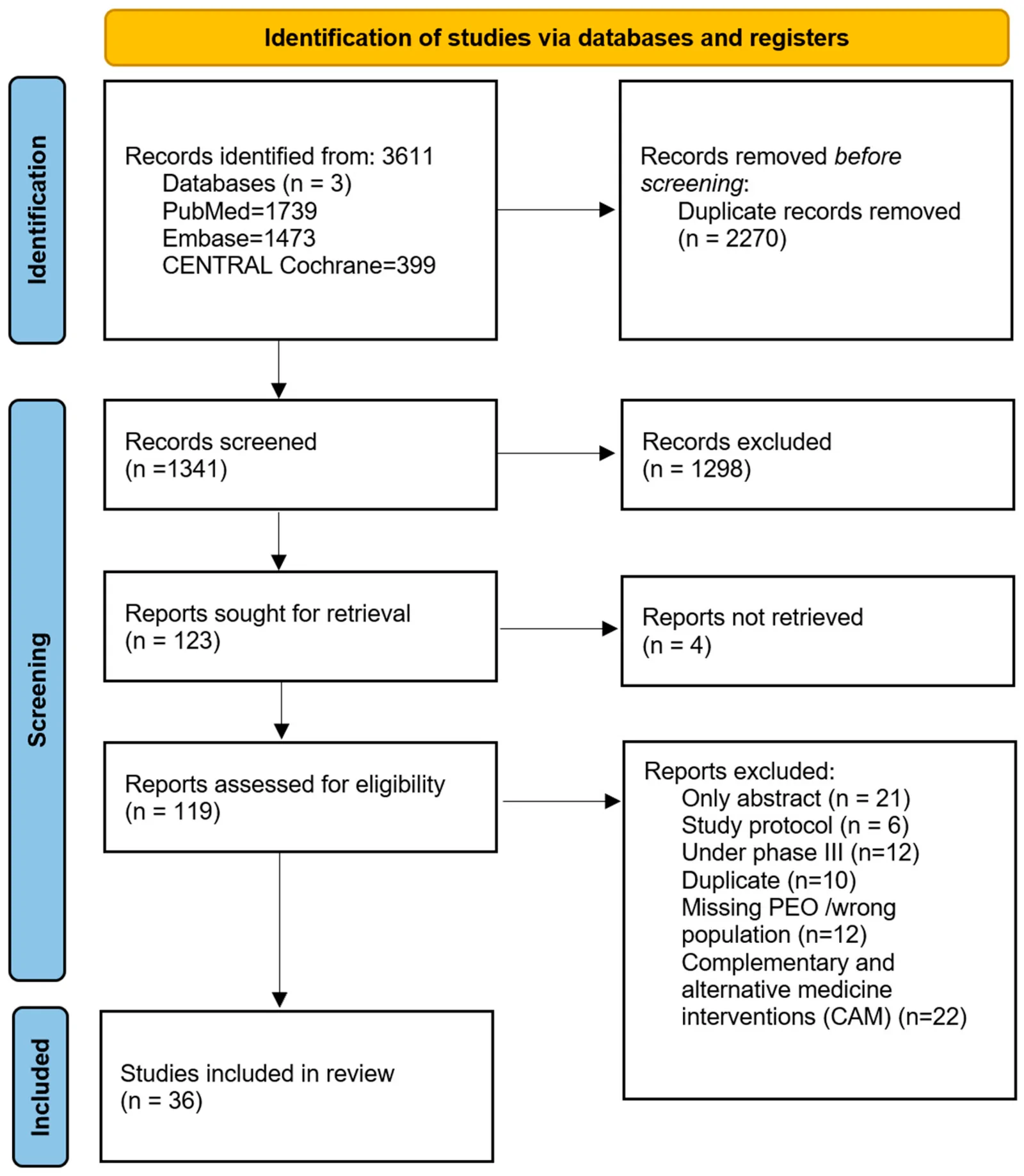
PRISMA 2020 flow diagram illustrating the identification, screening, eligibility assessment, and inclusion of studies in the systematic review.

This systematic review was conducted in accordance with the PRISMA 2020 recommendations [23] (Figure 1) and the methodological guidance of the Cochrane Handbook for Systematic Reviews of Interventions [24]. This review was designed to evaluate early perioperative interventions aimed at preventing prolonged postoperative ileus and improving gastrointestinal recovery after colorectal surgery in adults. The protocol was developed before data extraction and registered for PROSPERO (CRD420261307668) [25]. A systematic search was performed in PubMed/MEDLINE, Embase, and the Cochrane Central Register of Controlled Trials (CENTRAL). Eligible articles were restricted to studies published from 1 January 2000 onward. No language restrictions were applied. Only published full-text studies were considered.

### 2.2. Eligibility Criteria

Eligible studies included randomized controlled trials (RCTs), enrolling (P) adult patients aged 18 years or older undergoing colorectal surgery, either elective or emergency, for benign or malignant diseases. The (E) exposures of interest were early perioperative factors potentially associated with PPOI. These included preoperative factors, such as age, sex, BMI, smoking status, alcohol use, comorbidities, ASA score, chronic medication use, and laboratory parameters; intraoperative factors, such as surgical approach, operative duration, blood loss, transfusion, type of procedure, anastomotic characteristics, emergency versus elective surgery, and type of anesthesia; and early postoperative factors, such as opioid consumption, fever, early laboratory abnormalities, and early postoperative complications. The primary outcome (O) was clinically defined PPOI, according to the operational definition used by each individual study. PPOI was defined as persistence of gastrointestinal dysfunction beyond postoperative day 3–4, absence of flatus or stool by postoperative day 2–5, with presence of key clinical symptoms of inability to tolerate oral intake beyond expected recovery, need for nasogastric tube reinsertion, radiologic ileus without mechanical obstruction, paralytic ileus, or prolonged hospital stays attributed to ileus. Where a predefined PPOI definition was not provided, POI/PPOI reporting and surrogate measures of postoperative gastrointestinal recovery were extracted separately as secondary outcomes, including time to first flatus, time to first bowel movement or passage of stool, length of hospital stays, nasogastric tube reinsertion, postoperative complications, readmission, and reoperation.

Studies involving pediatric populations, non-colorectal abdominal surgery, or patients with pre-existing chronic intestinal pseudo-obstruction were excluded. Observational studies, non-randomized trials, case reports, case series, reviews, protocols, conference abstracts without full-text data and duplicate publications were excluded.

### 2.3. Search Strategy

The search strategy combined controlled vocabulary and free-text terms related to colorectal surgery, postoperative ileus, prolonged postoperative ileus, perioperative factors, and randomized study design and was performed on 16 February 2026. The search was structured around the following core concepts: adult colorectal surgery; postoperative ileus or prolonged postoperative ileus; perioperative, preoperative, intraoperative, or early postoperative predictors; and randomized clinical trials. The search identified 3611 records: 1739 from PubMed, 1473 from Embase, and 399 from CENTRAL. The complete electronic search strategies for PubMed/MEDLINE, Embase, and CENTRAL are provided in Supplementary Table S2.

### 2.4. Study Screening

All retrieved citations were imported into *Endnote 20* (Clarivate, 2013) for duplicate removal. After deduplication, 2270 duplicate records were excluded, leaving 1341 records for screening. The remaining records were screened by title and abstract by two independent co-investigators (C-S.H., A-A.H.) with *Rayyan* (Version 1.0, Qatar Computing Research Institute (QCRI), Qatar) [26] followed by full-text assessment of potentially eligible studies. Cohen’s kappa coefficient (k) was calculated to measure inter-rater reliability after each selection step. Conflicts were resolved by a third independent reviewer (I.C-M.). A total of 119 randomized clinical trials were assessed as potentially eligible, of which 36 reported relevant data for the predefined outcomes and were included in the final synthesis.

### 2.5. Data Extraction and Synthesis

Data were extracted independently by three reviewers (C-S.H., A-A.H., A-L.A) using a standardized data extraction form developed before analysis. Extracted data included first author, year of publication, country, study design, sample size, patient demographics, benign or malignant pathology, intervention or exposure, comparator where applicable, perioperative protocol, surgical approach, operative duration, blood loss, transfusion, stoma formation, anesthesia or analgesic strategy, bowel preparation, antibiotic regimen, and relevant laboratory parameters. Outcome data included the study definition of PPOI, rate of POI, rate of PPOI, time to first flatus, time to first bowel movement, length of hospital stay, need for nasogastric tube reinsertion, postoperative complications, readmission, and reoperation. Missing data were recorded as not reported. Effect estimates and 95% confidence intervals (CIs) were reported when provided by the original studies; when these were unavailable, group-level estimates and corresponding *p*-values were considered.

Studies were synthesized narratively according to perioperative phase and type of factor or intervention: preoperative, intraoperative, perioperative, postoperative, and mixed-phase strategies. The feasibility of meta-analysis was considered both across the overall evidence base and within clinically comparable intervention–outcome subsets. However, quantitative pooling was ultimately not performed because clinically and methodologically sufficiently homogeneous subsets could not be identified, owing to differences in intervention protocols, outcome definitions and assessment methods, and reporting formats. Outcomes were interpreted in relation to both direct PPOI reporting and surrogate markers of gastrointestinal recovery, including first flatus, first bowel movement, nasogastric tube reinsertion, and length of hospital stay. The direction of effect was categorized as beneficial, partially beneficial, or not beneficial according to the reported impact on PPOI or clinically relevant recovery outcomes. This classification was used solely for descriptive purposes and was not intended as a quantitative estimate of treatment efficacy. Studies were not weighted equally when interpreting the overall evidence; study size, risk of bias, consistency of findings, and GRADE certainty were also considered.

### 2.6. Risk-of-Bias Analysis and Certainty of Events

Risk of bias was assessed independently by three reviewers (C-S.H., A-A.H., A-L.A). For randomized clinical trials, the Cochrane Risk of Bias 2 tool was used [27]. The assessed domains included bias arising from the randomization process, deviations from intended interventions, missing outcome data, outcome measurement, and selection of reported results. The overall certainty of the evidence for each outcome was independently assessed using the GRADE methodology and GRADEpro GDT software, considering the risk of bias, inconsistency, indirectness, imprecision, and publication bias [28].

## 3. Results

### 3.1. Study Characteristics

Data were extracted from 36 randomized controlled clinical trials that met the aforementioned outcomes [29,30,31,32,33,34,35,36,37,38,39,40,41,42,43,44,45,46,47,48,49,50,51,52,53,54,55,56,57,58,59,60,61,62,63,64]. A clear distinction was made between clinically defined PPOI and surrogate measures of postoperative gastrointestinal recovery. Of the 36 included RCTs, 7 (19.4%) studies reported an operational definition POI/PPOI, of which only 5 [36,38,43,48,60] (13.9% of all included RCTs) applied a predefined time-based operational definition corresponding specifically to PPOI, whereas the remaining 2 used operational definitions that did not meet this more specific criterion. An additional 12 studies (33.3%) reported POI, PPOI, prolonged ileus, or paralytic ileus without sufficiently detailed diagnostic criteria, while the remaining 17 studies (47.2%) primarily evaluated gastrointestinal recovery through surrogate outcomes, including time to first flatus, time to first bowel movement, tolerance of oral intake, nasogastric tube reinsertion, or length of hospital stay. Among studies providing an explicit temporal threshold, the definition of prolonged gastrointestinal dysfunction varied considerably, ranging from POD2 to POD5.

The population characteristics of the included studies are reported in Table 1. Across the included studies, patient populations were largely comparable, with a predominance of malignant pathology and a mean age ranging between 55 and 75 years and a relatively balanced sex distribution.

Smoking status was mentioned in seven studies [36,37,38,42,43,51,53] with an incidence between 8 and 45.6%, with no relation found with PPOI incidence in intervention groups. No study reported information regarding alcohol consumption status of the patients.

Only six studies mentioned multiple comorbidities in patients enrolled [29,31,32,34,53,62]. Charlson comorbidity score was noted only by Nasseri et al. [37], which reported 4.3 ± 2.1 in the decaffeinated group and 3.5 ± 2.5 in the caffeinated group, both values higher than the warm water group who presented 3.03 ± 2.1.99.

In seven studies, previous abdominal surgery was reported [29,32,42,43,44,47,49], and four studies considered it an exclusion criterion [38,50,52,63].

### 3.2. Length of Hospital Stay and Clinic

Length of hospital stay varied substantially across studies, reflecting differences in patient populations, surgical approaches, perioperative pathways, and institutional discharge criteria. Therefore, absolute LOS values should not be compared directly across trials. Where intervention–control comparisons were available within individual RCTs, several multimodal and opioid-sparing strategies were associated with shorter hospitalization. These studies were predominantly conducted in mixed malignant/benign cohorts or elective colorectal cancer populations and reported earlier gastrointestinal recovery within individual randomized intervention–control comparisons. However, these improvements in recovery outcomes do not necessarily indicate a reduced incidence of clinically defined PPOI. Similarly, trials evaluating interventions such as prucalopride, coffee, and tibial nerve stimulation reported relatively short hospital stays in malignant or mixed cohorts. However, these absolute LOS values cannot independently establish intervention efficacy, and any potential benefit should be interpreted according to the within-trial randomized comparisons and the specific outcomes assessed.

In contrast, longer LOS, extending up to 9–16 days, was observed in studies evaluating interventions with limited or inconsistent efficacy on bowel function, including gum chewing, choline citrate, erythromycin, and certain laxative strategies.

Furthermore, studies involving diversion procedures or higher ASA scores tended to report longer LOS, reflecting increased perioperative risk and potentially higher susceptibility to PPOI. Conversely, trials excluding high-risk patients or implementing standardized perioperative pathways reported shorter LOS and improved recovery metrics.

### 3.3. Perioperative Protocol Particularities

**Figure 2:**
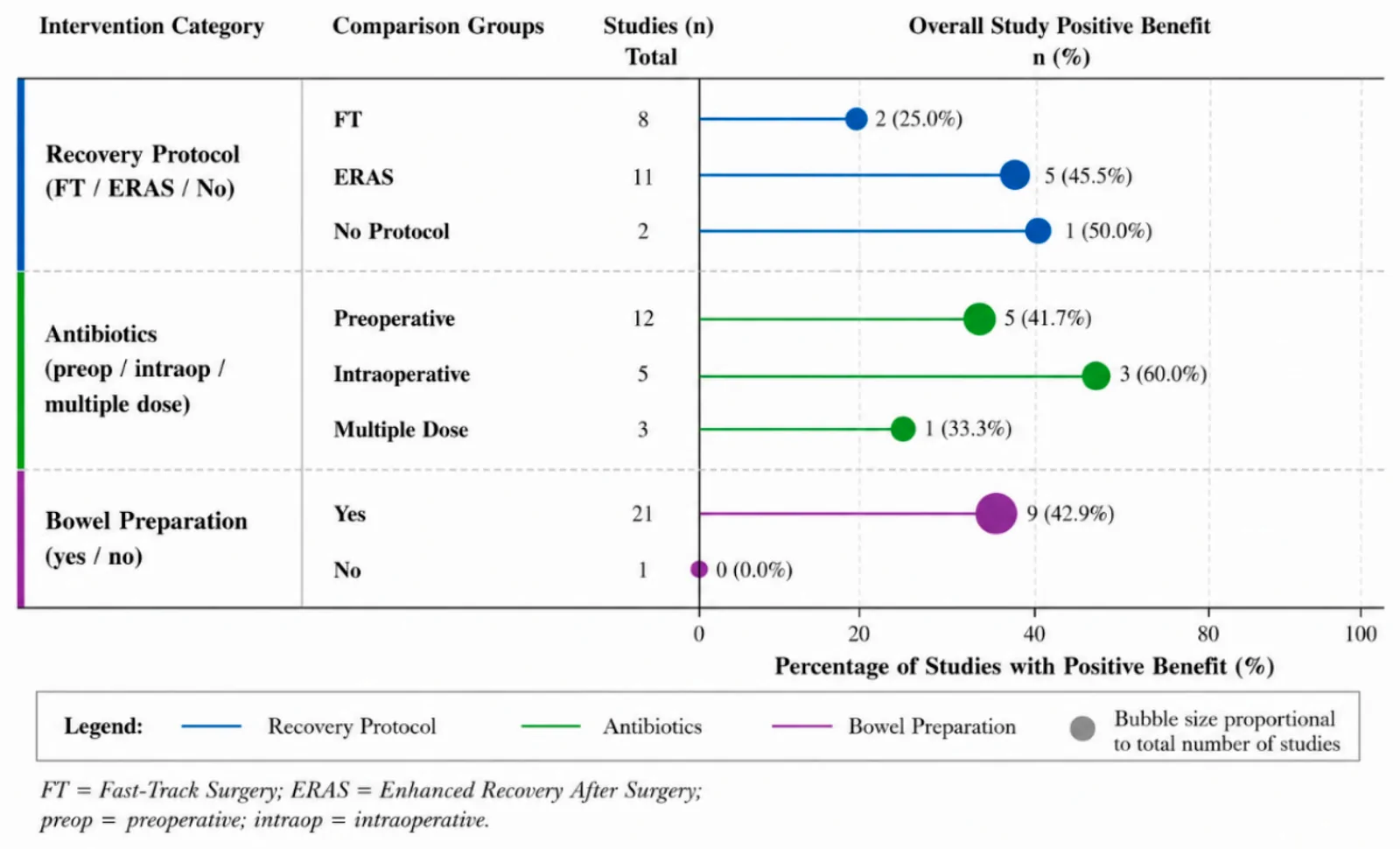
Descriptive mapping of perioperative protocol characteristics in studies evaluating postoperative ileus.

Perioperative protocol regarding recovery measurements, antibiotics and bowel preparation is registered in Figure 2. Structured perioperative pathways were present in several studies reporting favorable gastrointestinal recovery outcomes. Trials conducted within ERAS or fast-track pathways showed heterogeneous effects across the randomized interventions evaluated. Because ERAS or fast-track care generally represented the background perioperative pathway rather than the randomized intervention itself, these findings should be interpreted within the randomized intervention–control comparison of each individual trial and do not establish an independent effect of ERAS or fast-track care on PPOI or gastrointestinal recovery.

Antibiotic administration was most commonly reported as a component of background perioperative care rather than as the randomized intervention, and cross-study differences according to antibiotic exposure or timing should, therefore, be interpreted descriptively. An exception was the randomized trial by Espin-Basany et al. [62], in which 300 patients were allocated to three doses, one dose, or no oral antibiotic prophylaxis. In this trial, the randomized antibiotic regimens did not result in significant differences in the reported postoperative outcomes between groups. Accordingly, while this individual randomized comparison permits assessment of the specific antibiotic prophylaxis regimens evaluated, the broader evidence across the included RCTs does not establish an independent effect of antibiotic exposure or timing on PPOI or gastrointestinal recovery. Bowel preparation was incorporated into the perioperative protocol of most included studies [31,32,33,38,42,44,46,58,60], whereas one study [39] intentionally omitted bowel preparation. Because bowel preparation was generally not independently randomized, differences in gastrointestinal recovery between studies using different preparation protocols are descriptive and potentially confounded by the randomized intervention, surgical approach, patient characteristics, and other components of perioperative care. Therefore, these data do not establish an independent effect of bowel preparation on PPOI prevention.

### 3.4. Intraoperative Protocol Particularities

Across the included studies, operative characteristics varied according to the predominant surgical approach (Table 2); however, these cross-study differences are descriptive because surgical approach was not independently randomized in most trials. An exception was Gervaz et al. [44], in which patients undergoing elective sigmoid resection for diverticulitis were randomized to laparoscopic or conventional open sigmoid colectomy. Accordingly, any effect of surgical approach can be interpreted from this within-trial randomized comparison, whereas differences between laparoscopic, open, and mixed cohorts across the remaining studies cannot establish an independent effect of minimally invasive surgery on PPOI or gastrointestinal recovery. Blood loss ranged from 18.4 to 177.1 mL in laparoscopic studies, compared with 111 to 260 mL in open procedures and up to 521 mL in mixed cohorts. Only one [46] study reported intraoperative transfusion rate, within laparoscopic cohort, with a maximum transfusion rate of 8%. Operative duration was variable across all approaches, ranging from 102.0 to 231.2 min in laparoscopic studies, 108.0 to 271.6 min in open procedures, and 145 to 244.2 min in mixed cohorts. Stoma formation was reported in 1 [52] of 10 laparoscopic studies, compared with 3 [34,47,56] of 13 open studies and 3 [36,43,48] of 14 mixed cohorts.

### 3.5. Study Outcomes and Definitions of PPOI

**Figure 3:**
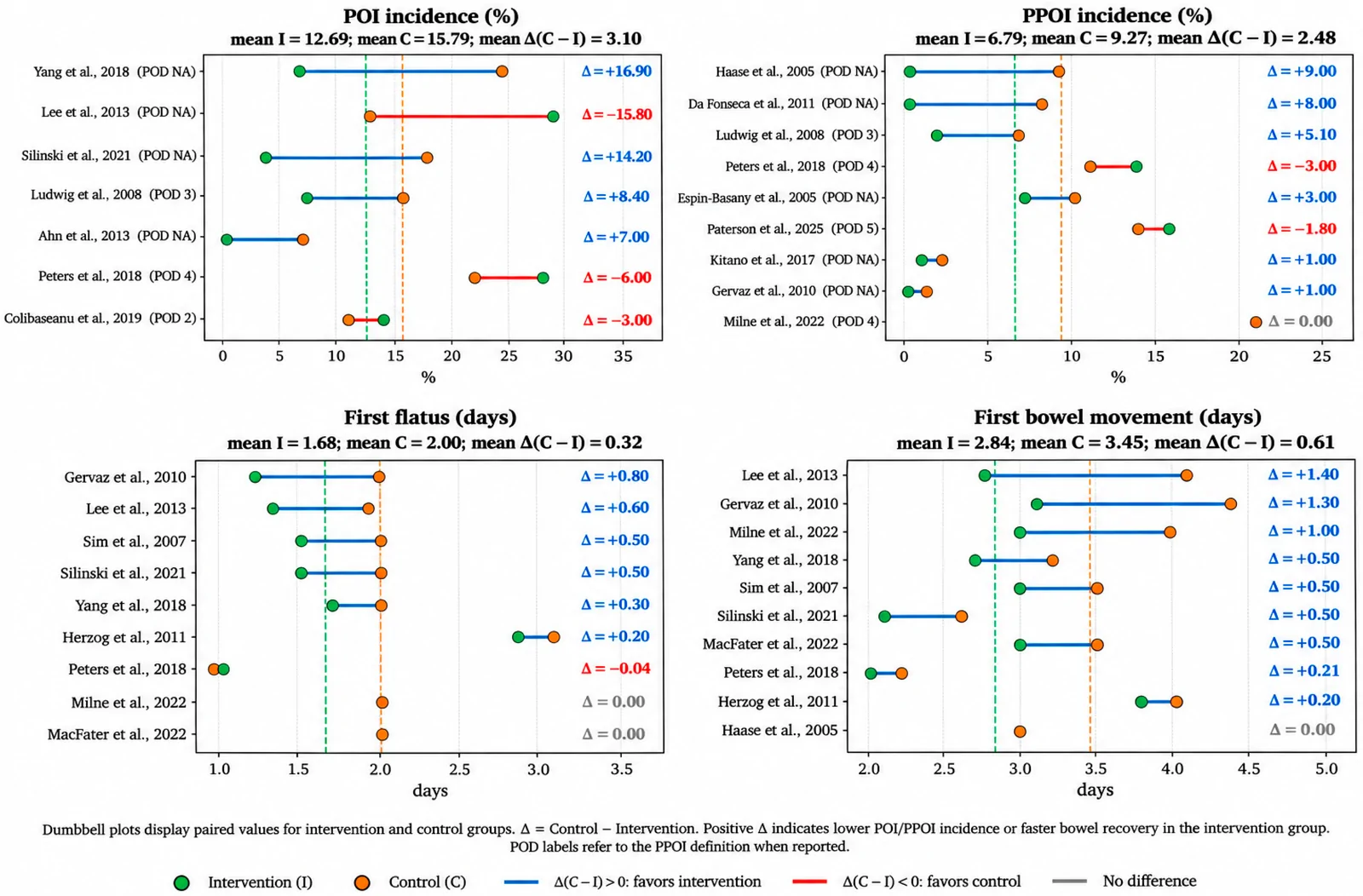
Study-level reporting of POI/PPOI definitions, POI/PPOI incidence, and surrogate gastrointestinal recovery outcomes [30,34,36,38,43,44,45,48,50,51,52,54,55,56,57,58,62].

Our primary outcome was prolonged postoperative ileus. Secondary outcomes included the study of postoperative ileus rates, the rate of prolonged postoperative ileus, length of hospital stay, need for nasogastric tube reinsertion, time to first flatus, and time to first bowel movement in relation to different interventions. Importantly, outcomes presented in Figure 3 should not be interpreted as equivalent measures of PPOI, as most represent POI or surrogate markers of gastrointestinal recovery rather than clinically defined PPOI.

Figure 3 provides information on the studies in which the postoperative day from which prolonged postoperative ileus was defined, the rate of postoperative ileus, or the rate of prolonged postoperative ileus was explicitly reported. The dumbbell plots particularly emphasize the variability in treatment effects between studies. Favorable differences between randomized intervention and control groups were observed were observed in selected studies [30,38,50,58], suggesting potential benefits of specific pharmacological, analgesic, nutritional, and non-pharmacological strategies on the gastrointestinal recovery outcomes assessed. However, improvements in surrogate recovery measures should not be interpreted as evidence of prevention of clinically defined PPOI unless this outcome is explicitly defined and evaluated. In contrast, several studies demonstrated minimal differences or even outcomes favoring control groups [43,52].

Only three [43,50,60] studies explicitly provided the corresponding results for PPOI definition, POI and PPOI incidence. But, despite the fact that they provided information for all three aforementioned categories, comparative information between groups was not fully provided.

Another important observation is the marked inconsistency in PPOI definitions, ranging from POD2 to POD5, which limits direct comparison across studies. Other terms and expressions used for occurrence of prolonged postoperative ileus were prolonged time to defecation, passage of stool, paralytic ileus, time to flatus, prolonged length of stay due to ileus, postoperative ileus-related morbidity, complications of postoperative ileus. Among the studies that explicitly defined the postoperative time threshold for prolonged postoperative ileus, postoperative day 3 was the most commonly used criterion for diagnosis [33,50,61,63,64].

Also, a methodological inconsistency in reporting outcomes is highlighted. Some studies reported means ± standard deviation, whereas others used medians and ranges. Outcome measures were expressed using different units, including hours versus postoperative days, limiting the possibility of direct quantitative comparison or pooled statistical analysis. Furthermore, several studies lacked complete data regarding either POI/PPOI incidence or bowel recovery parameters.

Da Fonseca et al. [58] reported an overall benefit of early feeding strategy and a benefit in first flatus day 1.5 ± 0.5 versus 2.0 [±0.7] days. In this study, the surgeons discharged the patients (11 of 24 in intervention group) even before first bowel movement, so this was not a reliable report for analyzing the link between prolonged postoperative ileus and first bowel movement without mentioning the rate of readmission, but they highlighted an important difference between groups in mean length of hospital stay, with 4.0 (±3.7) days in the intervention group and 7.6 (±8.1) days in control. Although the difference in hospital stay represents a clinically relevant outcome, the findings should be interpreted cautiously given the methodological limitations and overall high risk of bias of this trial primarily due to post-randomization exclusions and deviations from the intended intervention, which may have affected the comparability of the randomized groups and the estimated intervention effect.

### 3.6. Effects of Perioperative Interventions

**Figure 4:**
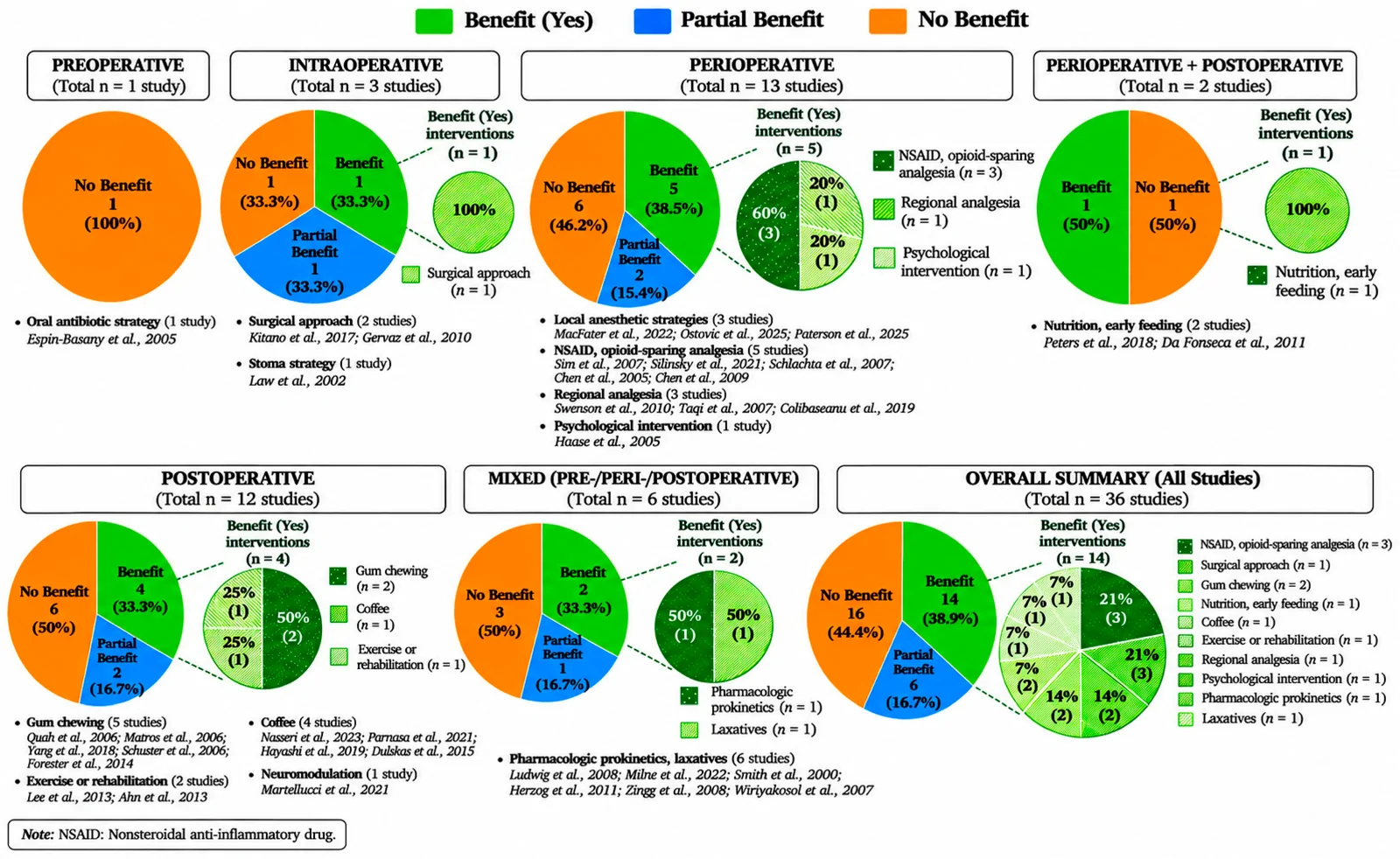
Descriptive mapping of perioperative interventions evaluated for postoperative gastrointestinal recovery, categorized according to intervention timing and type. The figure summarizes the distribution of interventions across the included randomized controlled trials and does not represent a quantitative comparison of treatment efficacy or evidence of prevention of clinically defined prolonged postoperative ileus (PPOI) [29,30,31,32,33,34,35,36,37,38,39,40,41,42,43,44,45,46,47,48,49,50,51,52,53,54,55,56,57,58,59,60,61,62,63,64].

Figure 4 provides a descriptive mapping of the included evidence according to intervention category, perioperative timing, and the reported direction of effects on postoperative bowel recovery. Considerable heterogeneity was observed across studies, with reported outcomes ranging from favorable effects [30,33,34,38,42,44,46,49,50,55,58,61] to partial improvements or no statistically significant differences. The evidence map identified studies addressing preoperative [62], intraoperative, perioperative, and postoperative strategies, encompassing surgical and stoma-related approaches, analgesic regimens, early feeding, gum chewing [38,49] coffee-based interventions [32,42], exercise [55], neuromodulation [39] and pharmacologic interventions [33,50]. For gum chewing, Yang et al. [38] reported POI in 7.0% of patients receiving xylitol gum compared with 23.9% of controls (*p* = 0.028), together with a significantly shorter time to first defecation (*p* = 0.0001). However, other postoperative outcomes, including abdominal distension (23.3% vs. 28.3%; *p* = 0.590), nausea (13.9% vs. 23.9%; *p* = 0.232), and vomiting (11.6% vs. 19.6%; *p* = 0.304), did not differ significantly between groups. Within perioperative care, several studies of NSAID-based or opioid-sparing analgesia reported favorable gastrointestinal recovery outcomes [30,34,61], whereas findings for local anesthetic and regional analgesic strategies were variable [46,54]. Among opioid-sparing strategies, Chen et al. [61] reported an 18.3% reduction in postoperative morphine consumption with ketorolac plus morphine compared with morphine alone, accompanied by earlier first bowel movement and passage of flatus.

In their regression analysis, patients receiving morphine alone had a 5.25-fold higher risk of prolonged bowel immobility than those receiving morphine plus ketorolac, supporting a potential effect of the randomized analgesic strategy on gastrointestinal recovery. Similarly, postoperative and mixed-phase interventions showed heterogeneous patterns of gastrointestinal recovery across the evaluated outcomes. Parnasa et al. [42] found that caffeine shortened the median time to first bowel movement from 69.8 (IQR 60.4–113.7) h with placebo to 51.3 (40.5–86.1) h (*p* = 0.012). However, the differences in first flatus (51.3 vs. 57.3 h; *p* = 0.290), tolerance of solid food (93.6 vs. 109.6 h; *p* = 0.581), and hospital stay (6 vs. 7 days; *p* = 0.299) were not statistically significant. Interpretation of the isolated improvement in first bowel movement should also consider the methodological concerns related to post-randomization exclusions and incomplete outcome data. In contrast, Nasseri et al. [37] found no clear effect on time to first bowel movement. Compared with warm water, the adjusted difference was −0.32 days for decaffeinated coffee (95% CI −0.99 to 0.36; *p* = 0.36) and −0.10 days for caffeinated coffee (95% CI −0.74 to 0.55; *p* = 0.77). This mapping is intended to characterize the distribution and direction of the available evidence rather than to establish comparative efficacy between interventions, as differences in sample size, intervention design, perioperative pathways, outcome definitions, and risk of bias preclude direct comparison based on the number of studies reporting statistically significant effects. Accordingly, favorable findings from individual trials were interpreted in the context of their methodological limitations and should not be considered equivalent in evidential weight.

### 3.7. Analgesic Strategies and Gastrointestinal Recovery

Postoperative outcomes varied according to analgesic strategy (Table 3). Readmission was noted only shortly after discharge, in relation to the period of hospitalization. Readmission for planned stoma reversal and restoration of intestinal continuity within the 30- or 90-day follow-up period were not considered PPOI-related readmissions, as these represented planned subsequent surgical procedures rather than complication of the index hospitalization.

Opioid-based or multimodal opioid regimens demonstrated wide variability in gastrointestinal recovery, with time to first flatus ranging from 1.1 to 4.1 days [29] and first bowel movement from 0.8 to 5.2 days [29], alongside higher reoperation rates 0–3 [44] and reported nasogastric tube reinsertion 3.3–18% [56]. NGT reinsertion was generally low but, when reported, appeared higher in studies with delayed bowel recovery or less effective interventions such as prokinetic/laxative [31,48] or psychological intervention groups [56], suggesting that persistent ileus requiring decompression correlates with poorer gastrointestinal recovery.

In contrast, non-opioid and multimodal analgesic strategies were associated with more consistent and favorable recovery profiles in some trials, with time to first flatus within 1.5–2.0 days [30,37,46] across studies and first bowel movement within 2.0–3.0 days [30,37,46] across studies, as well as lower complication rates. Regional analgesia, such as thoracic epidural catheter, showed intermediate results, with relatively early return of bowel function but limited reporting on prolonged postoperative ileus, more important results highlighted in association with non-opioid analgesia (use of flurbiprofen or acetaminophen), with time to first flatus in 0.3/[0.02–1.9] days [32]. This evidence was also sustained by Taqi et al. [46], who reported earlier gastrointestinal recovery with thoracic epidural analgesia than with patient-controlled opioid analgesia. Median time to passage of flatus was 2 days (95% CI 1.63–2.59) versus 3 days (95% CI 2.48–3.52; *p* = 0.0061), while time to first bowel movement was 2.78 ± 1.42 versus 4.04 ± 1.46 days (*p* = 0.0027). These differences should nevertheless be interpreted with some caution because of limitations in allocation concealment and the inherent difficulty of blinding the analgesic intervention. Overall, these findings support potentially favorable effects of selected randomized analgesic strategies on gastrointestinal recovery; however, these effects should be attributed to the randomized analgesic strategy as a whole rather than specifically to opioid reduction, which was not independently randomized in these trials.

### 3.8. Laboratory Parameters and Biological Markers

Particular attention was also given to the analysis of laboratory parameters; however, limited data were available regarding key biological markers involved in predicting the risk of prolonged postoperative ileus (Table 4). Only a small number of studies reported values for albumin (pre- and postoperative) [46,62], C-reactive protein [41,54], leukocyte count [41,54], hematocrit [62] and potassium [33].

Analysis of laboratory parameters revealed limited reporting across the included studies [33,41,46,54,62]. Preoperative lower hematocrit levels are increasingly recognized in surgical patients as a potential factor for postoperative complications and prolonged hospital stay and highly associated with need for blood transfusions in 7–9% [62,65].

**Table 4:** Laboratory analysis in included studies.

Study (Author, Year)	Timing	Parameter	Value in Each Intervention Group (s); Value in Control Group	Cut-Off/Risk Threshold
Espin-Basany et al., 2005 [62]	Preoperative	Hematocrit	37.2%/37.3%; 37.8%	Not defined
Espin-Basany et al., 2005 [62]	Preoperative	Albumin	3.63g/dL/3.60 g/dL; 3.61 g/dL	<3.915 g/dL (Liang et al., 2020) [21]
Taqi et al., 2007 [46]	Preoperative	Albumin	4.3g/dL/(4.098-4.327) *; 4.2g/dL/(4.050-4.325) *	<3.915 g/dL (Liang et al., 2020) [21]
Wiriyakosol et al., 2007 [33]	Postoperative	Potassium	3.78 ± 0.44 mmol/L	<3.5 mmol/L (Chu et al., 2022) [66]
MacFater et al., 2022 [54]	Postoperative	WBC	11.7 ± 2.6; 10.7 ± 2.8 × 10^9^/L	Not defined
Ostović et al., 2025 [41]	Postoperative	WBC	~12–13 × 10^9^/L; 12 ± 0.77 × 10^9^/L	Not defined
MacFater et al., 2022 [54]	Postoperative	CRP	92.0 ± 55.9 vs. 73.3 ± 46.1 mg/L	Not defined
Ostović et al., 2025 [41]	Postoperative	CRP	97 ± 6.8/104 ± 6.8/97 ± 9.6 mg/L	Not defined

WBC—white blood cell; s—study results; CRP—C-reactive protein; * mean/range.

Preoperative albumin levels ranged from borderline low values 3.60–3.63 g/dL [62] to normal levels 4.3 g/dL [46], with cut-off values below 3.915 g/dL [21] previously associated with increased risk of prolonged postoperative ileus (PPOI). Postoperatively, potassium levels were reported in one study [33] (3.78 ± 0.44 mmol/L), while values in the range of 3.0–3.5 mmol/L or lower have been described in the literature as impairing smooth muscle and slow gastrointestinal motility [66].

Postoperative inflammatory markers were reported in only a small subset of the included trials. MacFater et al. [54] and Ostović et al. [41] reported postoperative elevations in WBC and CRP, but no significant differences were observed between randomized intervention groups. Therefore, these trial data do not demonstrate an association between the magnitude of the postoperative inflammatory response and PPOI, nor do they establish inflammation as a mediator of the observed intervention effects.

### 3.9. Risk of Bias and Certainty of Events

**Figure 5:**
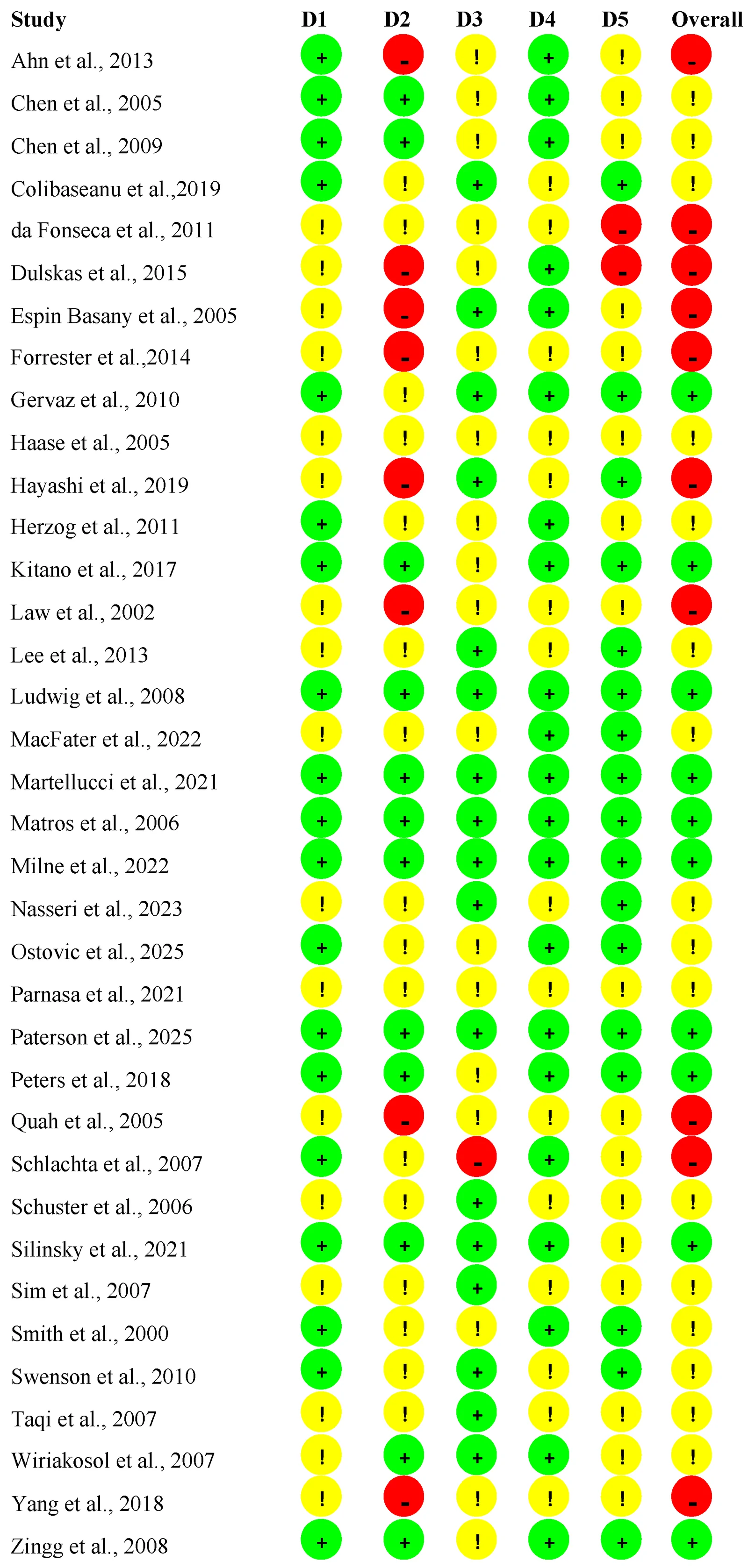
Risk-of-bias assessment of the included randomized controlled trials using the Cochrane RoB 2 tool. Green (+) low risk of bias; yellow (!) some concerns; red (−) high risk of bias. D1—risk of bias arising from the randomization process; D2—risk of bias due to deviations from intended interventions; D3—missing outcome data; D4—risk of bias in measurement of the outcome; D5—risk of bias in selection of the reported result [29,30,31,32,33,34,35,36,37,38,39,40,41,42,43,44,45,46,47,48,49,50,51,52,53,54,55,56,57,58,59,60,61,62,63,64].

Overall, the included randomized controlled trials demonstrated moderate methodological quality, with most studies classified as having some concerns rather than high risk of bias (Figure 5, Table 5). The randomization process was generally adequate, although reporting of allocation concealment was frequently incomplete.

Together, the clinical and methodological heterogeneity across studies supports the overall MODERATE certainty rating obtained using the GRADEpro approach (Table 6).

The most common limitation was related to deviations from intended interventions, largely due to lack of blinding in perioperative because open-label interventions such as gum chewing, coffee consumption, or epidural analgesia made participant awareness unavoidable and difficult to mask despite otherwise-standardized ERAS protocols in several studies [37,38,40,46,47].

Another major and recurrent methodological issue involved exclusions after randomization and failure to apply strict ITT analysis. Several studies analyzed fewer patients than initially randomized, often due to protocol deviations, postoperative complications, ICU admission, conversion to open surgery, or intervention non-adherence. Yang et al. [38] randomized 110 patients but analyzed only 89, while Zingg et al. [31] randomized 200 participants but analyzed only 169. Similar post-randomization losses were observed in Schlachta et al. [35], Ostovic et al. [41], Parnasa et al. [42], Dulskas et al. [53], Forrester et al. [63], Gervaz et al. [44], and Ahn et al. [55]. These exclusions raise the possibility that treatment effects were influenced by selective removal of patients with more complicated postoperative courses.

Missing outcome data and attrition bias were additional recurring concerns. Studies, such as Yang et al. [38], Zingg et al. [31], Parnasa et al. [42], Schlachta et al. [35], and Smith et al. [29], reported substantial losses to follow-up or exclusions after enrollment. In several cases, excluded patients experienced postoperative complications such as anastomotic leaks, ICU admission, or protocol intolerance, suggesting that missingness may have depended on the true clinical outcome and, therefore, increased the risk of attrition bias.

Selection bias related to imperfect allocation concealment was also frequently identified, particularly in older trials using sealed-envelope or manually handled randomization methods without adequate methodological detail [33,34,47,49] and studies where envelope randomization or card-pull systems were insufficiently described [46]. Additionally, some studies demonstrated baseline imbalances that may have influenced bowel recovery independently of the intervention itself, such as the markedly higher morphine consumption in controls reported [33].

Protocol deviations and incomplete adherence further contributed to heterogeneity between studies. MacFater et al. [54] reported frequent protocol breaches, including catheter dislodgement and conversion to open surgery, while Lee et al. [52] documented incomplete compliance with postoperative rehabilitation components. Variability in perioperative opioid administration, analgesic strategies, ERAS implementation, or postoperative supportive care was also noted [34,38,49], potentially confounding bowel recovery outcomes.

Despite these limitations, several studies [30,31,36,39,43,44,45,48,50,59] demonstrated consistently strong methodological quality, incorporated rigorous randomization methods, placebo or sham controls, double blinding, standardized ERAS pathways, and predefined ITT analyses. These trials generally exhibited lower risk of bias and, therefore, provide greater confidence in their reported intervention efficacy comparisons.

The certainty of evidence was evaluated using the GRADE approach for the principal outcomes of postoperative gastrointestinal recovery (Table 6). Evidence for PPOI incidence, derived from 10 randomized controlled trials including 2894 participants, was rated as low certainty because of very serious inconsistency between studies. The certainty of evidence for time to first flatus (28 trials; 2530 participants) and time to first bowel movement (27 trials; 2318 participants) was rated as very low, reflecting concerns regarding risk of bias, inconsistency, and imprecision. Evidence for nasogastric tube reinsertion, reported in 11 randomized trials including 2343 participants, was judged to be of moderate certainty, with downgrading for imprecision. Similarly, evidence for length of hospital stay, evaluated in 26 randomized trials involving 3078 participants, was considered moderate certainty, with one level of downgrading because of methodological limitations in the included studies.

## 4. Discussion

Postoperative ileus (POI) is a multifactorial and transient impairment of gastrointestinal motility that remains one of the most common complications following abdominal surgery.

This systematic review synthesizes evidence from 36 randomized controlled trials evaluating perioperative interventions in relation to PPOI and postoperative gastrointestinal recovery after colorectal surgery. Importantly, although seven trials (19.4%) reported an operational definition of POI/PPOI, only five (13.9% of all included trials) applied a predefined time-based definition corresponding specifically to PPOI. Overall, the findings highlight substantial heterogeneity in study design, definitions, and outcome reporting, which limits direct comparability across trials. Despite this variability, individual randomized trials identified potentially favorable effects of selected perioperative interventions on gastrointestinal recovery outcomes; however, the magnitude and consistency of these effects varied across trials and endpoints. In contrast, single-modality interventions demonstrate variable and often limited effects. Importantly, PPOI is considered a multifactorial complication involving the interplay between surgical stress, patient-related factors, and perioperative management; however, the independent contribution of these factors cannot be determined from the present RCT evidence.

The GRADE assessment demonstrated substantial variation in the certainty of evidence across the evaluated outcomes. Although PPOI incidence is the most clinically relevant endpoint, confidence in the available evidence remained limited owing to considerable heterogeneity in intervention strategies, perioperative management, and PPOI definitions, resulting in a low-certainty rating. Even lower certainty was observed for time to first flatus and first bowel movement, outcomes that were frequently reported using heterogeneous definitions and statistical measures and are inherently influenced by subjective assessment and local postoperative care protocols. In contrast, nasogastric tube reinsertion and length of hospital stay were supported by moderate-certainty evidence, suggesting greater consistency across studies despite remaining methodological limitations. Nevertheless, length of hospital stay should be interpreted cautiously because it reflects not only gastrointestinal recovery but also postoperative complications, discharge criteria, institutional practices, and implementation of enhanced recovery pathways. Moreover, return of bowel function may not represent an absolute prerequisite for hospital discharge in appropriately selected patients. The EuroSurg Collaborative prospective multicenter study of patients undergoing elective colorectal resection found that discharge before return of bowel function was not associated with an increased risk of 30-day readmission or postoperative complications [67]. These findings further emphasize that length of hospital stay and return of bowel function should not be considered interchangeable measures of postoperative gastrointestinal recovery and that discharge decisions may appropriately incorporate broader clinical recovery criteria rather than bowel function alone.

An important distinction should also be made between statistical significance in surrogate markers of gastrointestinal recovery and clinically meaningful postoperative benefit. Outcomes such as time to first flatus or first bowel movement are useful indicators of gastrointestinal motility but do not necessarily translate into a reduction in clinically defined PPOI, length of hospital stay, readmission, postoperative complications, or healthcare resource utilization. This distinction is illustrated by some of the included trials, in which statistically significant improvements in individual bowel-recovery endpoints were not accompanied by significant changes in length of stay. For example, Parnasa et al. [42] reported an approximately 18 h earlier first bowel movement with postoperative caffeine administration, whereas no significant reduction in hospital stay was observed. Therefore, relatively small or isolated improvements in surrogate recovery endpoints should not be interpreted as evidence of clinically meaningful benefit unless accompanied by improvements in patient-centered or healthcare outcomes. Future trials should prioritize predefined clinical PPOI endpoints and report outcomes such as length of stay, readmission, complications, and healthcare utilization alongside gastrointestinal recovery markers.

Overall, these findings indicate that current evidence supports several potentially beneficial perioperative interventions, but differences in study methodology and outcome reporting continue to limit the certainty of conclusions and reinforce the need for standardized PPOI definitions and harmonized outcome measures in future randomized trials.

A formal meta-analysis was not performed because of the substantial heterogeneity identified across the included studies. This heterogeneity emerged at multiple levels, including clinical heterogeneity (differences in patient populations, surgical approaches, perioperative care pathways, and interventions protocols), methodological heterogeneity (variations in study design, randomization procedures, blinding strategies, and outcome assessment), definitions of prolonged postoperative ileus and reporting heterogeneity. In particular, postoperative ileus and prolonged postoperative ileus were inconsistently defined, with studies variably reporting outcomes, such as time to first flatus, time to first bowel movement, GI-2/GI-3 recovery, tolerance of oral diet, nasogastric tube reinsertion, or overall gastrointestinal recovery. The lack of standardization in reporting data regarding precise rate of PPOI definition, POI incidence and PPOI incidence complicates direct comparison of outcomes and likely contributes to the variability in reported incidence rates. Furthermore, perioperative management protocols differed considerably between studies, especially regarding ERAS implementation, analgesic regimens, minimally invasive techniques, and postoperative feeding strategies. The feasibility of quantitative synthesis was also assessed within individual intervention categories and outcome-specific subsets. Meta-analysis was considered when multiple trials evaluated clinically comparable interventions and reported the same outcome using sufficiently compatible definitions and assessment methods. However, even within these subsets, important differences remained in intervention protocols, timing and dose, perioperative pathways, PPOI definitions, outcome assessment time points, and statistical reporting. Given these important differences, pooling the data quantitatively was considered methodologically inappropriate and potentially misleading. Therefore, a structured narrative synthesis was preferred to provide a more accurate and clinically meaningful interpretation of the available evidence.

A major concern arises from the lack of clear distinction between the terminology used across studies, such as “prolonged postoperative ileus” [34,36,37,43,48,55,57,60] and “postoperative ileus” not further specified [31,32,38,46,51,52,53,61,62,64] or using different terms and expressions like “complication of POI” [50], “gastrointestinal recovery 2 (GI2) delay” [36] or describing directly the installation of different symptoms that are specific to diagnose prolonged postoperative ileus without exactly mention the terms, such as “prolonged nausea or vomiting” [29,40] or “prolonged delay in first flatus or first bowel movement or bowel function” [44,49,63], “need for nasogastric tube reinsertion” [31,35,37,40,42,46,49,54,56], “prolonged hospital stay due to ileus” [31,33,42,43,49,50,59] or “need to readmission due to ileus” [50,59], which can be confusing and easy to misinterpret [4], issues that have been discussed already in previous systematic reviews and global surveys [4]. All of these findings are suggestive of prolonged postoperative ileus, although the condition was neither explicitly named nor formally classified as such. Standardized definitions and accurate documentation of PPOI are also important for consistent clinical communication and quality assessment [68,69].

Differences in reported PPOI incidence may reflect variations in definitions rather than true differences in treatment effects. From a GRADE perspective, such definitional and outcome heterogeneity may contribute particularly to inconsistency and, where outcome constructs differ substantially from the clinically relevant PPOI endpoint, to indirectness, thereby reducing confidence in the overall certainty of evidence. Standardization of PPOI diagnostic criteria and core outcome reporting is, therefore, essential to improve comparability between future trials and strengthen the certainty of evidence.

No clear direct association was identified between underlying pathology and PPOI risk. Both malignant-only cohorts (e.g., colorectal cancer) and mixed malignant/benign populations demonstrated similar ranges of LOS and recovery outcomes, aspects also identified in similar studies [37,48].

Longer hospital stay was more commonly observed in studies evaluating interventions with limited or inconsistent effects on postoperative bowel recovery, including gum chewing, choline citrate, erythromycin, and certain laxative-based strategies. These studies frequently included heterogeneous patient populations, higher proportions of open procedures, and more complex underlying pathology, such as advanced malignancy or diverticular disease, factors that may increase surgical stress, bowel manipulation, and baseline risk of prolonged postoperative ileus [8,16]. However, studies including exclusively oncologic patients did not consistently report prolonged hospitalization, indicating that the present evidence does not support malignant disease alone as an independent determinant of delayed gastrointestinal recovery.

Across the included trials, PPOI and gastrointestinal recovery outcomes varied according to underlying pathology, surgical complexity, patient comorbidity, and perioperative management; however, because these characteristics were generally not independently randomized, their individual contributions to PPOI cannot be established from the present review.

Studies [44,46,55,58] conducted within structured perioperative pathways, particularly fast-track and ERAS programs, reported favorable effects on selected clinically relevant outcomes, including earlier first flatus, earlier first bowel movement, reduced duration of postoperative ileus, shorter hospital stay, or reduced need for nasogastric tube reinsertion [16,70,71]. However, these findings should be interpreted within the randomized comparison of each individual trial and do not establish an independent effect of the background perioperative pathway. These protocols usually incorporated multimodal analgesia, early feeding, early mobilization, avoidance of routine nasogastric tubes, and minimally invasive surgery; however, the independent contribution of these background components to gastrointestinal recovery cannot be determined from the included RCTs because they were not independently randomized [8,70,71,72]. Baseline adherence to ERAS pathways could potentially modify the apparent effect of individual interventions aimed at accelerating gastrointestinal recovery; however, this effect could not be formally evaluated in the included trials. In highly adherent ERAS settings, several mechanisms targeted by single interventions, including opioid minimization, early oral feeding, early mobilization, avoidance of routine nasogastric decompression, and minimally invasive surgery, are already incorporated into multimodal perioperative care. Consequently, the incremental benefit attributable to an additional single-target intervention may be attenuated compared with that observed under conventional perioperative care. This may partly explain why interventions such as gum chewing have shown limited additional benefit in some trials conducted within established enhanced recovery pathways, whereas selected pharmacological interventions may retain an incremental effect in specific settings. However, ERAS implementation and adherence were inconsistently reported across the included RCTs, and most studies did not provide quantitative adherence data or stratified treatment effects according to ERAS compliance [48,50]. Therefore, the present review cannot determine whether ERAS adherence formally modifies the efficacy of individual interventions, particularly given the inconsistent reporting of pathway adherence and other protocol deviations across trials. Future randomized trials should report adherence to individual ERAS components and evaluate interaction between baseline pathway adherence and treatment effect to distinguish true additive clinical benefit from improvements already achieved through multimodal perioperative care.

Previous abdominal surgery was reported in several studies, while others excluded such patients because prior surgical interventions may increase intra-abdominal adhesions, operative complexity, bowel manipulation, and postoperative inflammatory response, potentially influencing gastrointestinal recovery and confounding the diagnosis of prolonged postoperative ileus [8,72,73,74].

The favorable outcomes reported in some studies using preoperative antibiotics may reflect the fact that these regimens were frequently embedded within more comprehensive bowel preparation and perioperative optimization pathways.

Similarly, the apparent association between bowel preparation and positive outcomes should be interpreted cautiously. Most of the included studies used some form of bowel preparation, meaning that it was often part of standard colorectal perioperative care rather than an independently tested intervention [29,31,33,48,50,57]. The role of antibiotic administration should be interpreted according to whether it constituted the randomized intervention or merely a component of background perioperative care. In Espin-Basany et al. [62], 300 patients were randomized to receive three doses, one dose, or no oral antibiotic prophylaxis. The randomized comparison did not demonstrate significant differences in the reported postoperative outcomes between groups and, therefore, does not provide evidence that the specific oral antibiotic regimens evaluated independently improved postoperative gastrointestinal recovery. In the remaining trials, antibiotic administration was generally embedded within broader perioperative protocols; consequently, associations between antibiotic exposure and gastrointestinal recovery in these studies are descriptive and potentially confounded and cannot establish an independent effect on PPOI [74]. Similarly, the apparent association between bowel preparation and favorable outcomes should be interpreted descriptively. Most included studies used some form of bowel preparation as part of standard perioperative care rather than as an independently randomized intervention. Therefore, the available evidence does not establish an independent effect of bowel preparation on PPOI prevention or gastrointestinal recovery.

Perioperative interventions represented the most extensively investigated category, with several randomized trials evaluating NSAID-based and multimodal analgesic strategies, including those by Sim et al. [34], Silinsky et al. [30], Schlachta et al. [35], Chen et al. [64], and Chen et al. [61]. These strategies showed heterogeneous effects across gastrointestinal recovery outcomes; therefore, their findings should be interpreted according to the magnitude and precision of the intervention–control differences reported within individual trials rather than the number of studies reaching statistical significance. Importantly, the observed effects should be attributed to the randomized analgesic strategies rather than specifically to opioid reduction, which was not independently randomized in these trials. From a mechanistic perspective, reduced opioid exposure and attenuation of the postoperative inflammatory response represent plausible mechanisms through which some analgesic strategies may influence gastrointestinal recovery [71]; however, their independent contribution to PPOI cannot be established from these trials. Confidence in these findings nevertheless varies across trials because methodological limitations, including incomplete allocation concealment, lack of blinding, protocol deviations, and incomplete outcome data, affected some of the analgesic studies. Thoracic epidural analgesia, evaluated by Colibaseanu et al. [51], may influence postoperative gastrointestinal recovery through several mechanisms, including blockade of inhibitory sympathetic reflexes, improved pain control, and reduced systemic opioid requirement [16,75].

Minimally invasive surgery should be distinguished as a directly randomized intervention from its more frequent role as a background surgical characteristic across the included trials. Gervaz et al. [44] directly randomized patients undergoing sigmoid colectomy to laparoscopic versus conventional open surgery, allowing the effects of surgical approach to be interpreted within this specific randomized comparison. In the remaining studies, however, surgical approach was generally not independently randomized and often coexisted with differences in patient selection, operative complexity, and perioperative management. Therefore, the broader cross-study differences between laparoscopic, open, and mixed cohorts should be considered descriptive and cannot establish an independent protective effect of minimally invasive surgery against PPOI. The favorable findings reported for gum chewing by Quah et al. [47] and Yang et al. [38] can be explained by the “sham feeding” mechanism, which activates the cephalo-vagal reflex, stimulates digestive hormone secretion, and promotes restoration of intestinal peristalsis. Gum chewing was associated with improvements in selected gastrointestinal recovery outcomes in some individual trials [38,47]. For example, Yang et al. [16] reported a lower incidence of POI and earlier defecation (7.0% vs. 23.9%; *p* = 0.028), although several other postoperative outcomes did not differ between groups; however, the magnitude of this apparent intervention effect should be interpreted cautiously in light of methodological limitations because post-randomization exclusions (110 randomized, 89 analyzed), difficulty of blinding, the reliance on subjective gastrointestinal recovery endpoints and incomplete outcome data may have influenced the intervention–control comparison. However, its effectiveness appeared less pronounced in modern Enhanced Recovery After Surgery (ERAS) pathways, where early mobilization and feeding are already routinely implemented [71].

Coffee-based interventions produced heterogeneous findings across trials, with improvement in selected gastrointestinal recovery endpoints [37] but without consistent effects across outcomes. These findings should be interpreted cautiously where blinding was not feasible and outcomes relied on patient-reported or otherwise subjective measures of gastrointestinal recovery. Proposed mechanisms underlying the effects of coffee on gastrointestinal motility include neurohormonal stimulation involving gastrin and cholecystokinin release and adenosine receptor blockade [76]. Exercise and rehabilitation interventions showed potentially favorable effects on bowel recovery in selected trials; however, incomplete adherence to rehabilitation components in some studies limits confidence in the magnitude of the observed effect. Proposed mechanisms include enhanced parasympathetic activity, reduced inflammatory signaling, and improved intestinal perfusion [72].

Mixed interventions spanning the preoperative, perioperative, and postoperative periods included laxatives and prokinetic agents, as reported by Herzog et al. [57] and Ludwig et al. [50]. These interventions directly stimulate gastrointestinal motility through activation of the enteric nervous system and receptors involved in peristalsis; however, their effects on postoperative gastrointestinal recovery outcomes were inconsistent across trials, and the present evidence does not allow these differences to be attributed to independent effects of postoperative inflammation or opioid exposure.

Although early postoperative dysmotility is initially triggered predominantly by neurogenic mechanisms, involving transient disruption of enteric neuromuscular activity within the myenteric (Auerbach’s) and submucosal (Meissner’s) plexuses and resulting in functional motor inhibition, increasing evidence suggests that persistence of POI and its progression to prolonged postoperative ileus (PPOI) are largely sustained by an exaggerated inflammatory response [11,22]. In this context, although the present descriptive evidence map does not permit conclusions regarding the comparative efficacy of individual interventions, the distribution of reported effects across different perioperative strategies further illustrates the multifactorial nature of postoperative gastrointestinal dysfunction. Favorable outcomes were reported across interventions targeting different components of this pathophysiological process, including opioid exposure, analgesia, enteral stimulation, mobilization, and gastrointestinal motility. This pattern is compatible with the transition from an early neurogenic phase to a more sustained inflammatory response following surgical manipulation and supports a multimodal approach to POI prevention and management. However, these observations should be interpreted as hypothesis-supporting rather than as evidence of superiority of any specific intervention or intervention category.

Postoperative inflammation represents a biologically plausible component of POI/PPOI pathophysiology, as supported by the mechanistic literature [4,22]. However, favorable gastrointestinal recovery observed with interventions that may also influence inflammatory pathways should not be interpreted as evidence that attenuation of postoperative inflammation independently mediated these effects.

In our review, only 2 of 36 studies [41,54], both evaluating local anesthetic strategies, reported quantitative postoperative inflammatory markers (CRP and WBC), and 1 study additionally reported IL-6 and IL-8 [41]. No significant between-group differences or clear relationship with PPOI were demonstrated, and, therefore, these RCT data do not establish an independent causal contribution of postoperative inflammation to PPOI. Maybe a more comprehensive study regarding inflammatory markers related to prediction of PPOI explored at 12 h, 24 h, 36 h postoperative could be at use in order to study activation of muscularis macrophages, enteric glial cells and recruitment of neutrophils and monocytes through adhesion molecules such as ICAM-1, resulting in increased production of IL-6, TNF-α, MCP-1, nitric oxide, and other mediators that impair enteric neuronal signaling and smooth muscle contractility, resulting in impaired intestinal motility [15,20,77]. In parallel, postoperative stress and inflammation disrupt intestinal barrier integrity through alterations in tight-junction proteins, particularly occludin and claudins, leading to increased intestinal permeability, bacterial translocation, and amplification of local inflammatory responses [20]. Experimental studies have demonstrated reduced expression of barrier-forming proteins such as occludin and claudin-1 together with increased expression of pore-forming claudin-2, changes that facilitate luminal antigen and lipopolysaccharide translocation and subsequent activation of TLR4-dependent inflammatory pathways [78].

Moreover, postoperative dysbiosis is characterized by a reduction in beneficial butyrate-producing bacteria and short-chain fatty acids, which normally preserve epithelial barrier function and exert anti-inflammatory effects. Loss of these protective microbiota-derived metabolites further compromises occludin-mediated barrier integrity, promotes immune activation, and contributes to prolonged intestinal dysmotility [79].

Preoperative lower hematocrit levels and anemia have become increasingly accepted in surgical patients due to the potential factors for postoperative complications and prolonged hospital stay and frequently associated with blood transfusions [65]. Malignant cohorts reported high incidence of blood transfusion possible due to occult or overt gastrointestinal bleeding, loss of blood during laborious surgical interventions, iron deficiency, neoadjuvant therapy side effects, iron deficiency [80].

Even mild–moderate postoperative hypokalemia with serum potassium [3.0–3.5]–[2.5–3.0] mmol/L [66] may also cause severe complications such as prolonged postoperative ileus due to its effects on smooth muscle membrane excitability and neuromuscular transmission. Potassium depletion may reduce intestinal peristalsis, contributing to postoperative bowel dysfunction and delayed gastrointestinal recovery in surgical patients. Similar findings have been reported in both the cardiovascular and emergency medicine literature, where hypokalemia was associated with gastrointestinal hypomotility, constipation, abdominal distension, and paralytic ileus [81,82,83,84].

Across the included randomized trials, the most frequent methodological limitations involved inadequate blinding, unclear allocation concealment, post-randomization exclusions, inconsistent adherence to interventions, incomplete intention-to-treat (ITT) analyses, and missing outcome data [73]. These flaws were particularly common in studies evaluating behavioral or perioperative interventions, such as gum chewing, coffee administration, epidural analgesia, rehabilitation, early feeding, or oral antibiotic strategies, where participant and caregiver blinding was often impractical.

Because many primary endpoints in postoperative ileus research, including first flatus, bowel movement, tolerance of diet, hunger, pain, or readiness for discharge, are subjective and behavior-dependent, the lack of blinding increased the risk of both performance and detection bias [71].

Accordingly, apparently favorable findings from trials affected by these methodological limitations should be interpreted cautiously, particularly when based on subjective recovery endpoints, small samples, incomplete outcome data, or analyses that deviated from the intention-to-treat principle. These limitations reduce confidence in the observed intervention effects; accordingly, apparently favorable findings from trials with substantial post-randomization exclusions, missing outcome data, protocol deviations, or post-randomization exclusions/incomplete ITT analyses were given less interpretative weight than findings from trials with more robust methodological conduct.

### 4.1. Strengths and Limitations

This systematic review has several strengths: it focuses exclusively on a large number of RCTs, providing a high level of evidence, and adopts a comprehensive perioperative perspective, integrating preoperative, intraoperative, and postoperative interventions within a unified mechanistic framework. Additionally, the analysis of PPOI definitions across studies represents a key contribution, highlighting an important source of heterogeneity often overlooked in previous reviews.

However, several limitations should be acknowledged. Substantial heterogeneity was present in study design, patient populations, interventions, and outcome reporting, which precluded quantitative synthesis. Importantly, most included trials were designed to evaluate interventions rather than prognostic associations, limiting the ability to identify independent risk factors for PPOI. Importantly, only 7 of the 36 included RCTs (19.4%) reported an operational definition of POI/PPOI, and only 5 of these (13.9% of all included RCTs) applied a predefined time-based definition corresponding specifically to PPOI. Most trials, therefore, relied on less clearly defined POI/PPOI outcomes or surrogate measures of gastrointestinal recovery. This substantially limits the extent to which findings across the entire evidence base can be interpreted as evidence for prevention of clinically defined PPOI. Finally, methodological limitations in several trials, including post-randomization exclusions, incomplete outcome data, lack of blinding, unclear allocation concealment, protocol deviations, and departures from intention-to-treat analysis, reduce confidence in some apparently favorable intervention effects and were, therefore, incorporated into the interpretation of individual findings.

### 4.2. Implications for Practice and Research

The findings identify several potentially promising perioperative strategies associated with improved gastrointestinal recovery, including multimodal analgesic approaches, early mobilization, and early enteral stimulation; however, the available evidence does not establish their independent efficacy for preventing PPOI. Clinically, PPOI should be regarded as a multifactorial and potentially modifiable complication rather than a condition driven by isolated factors, reflecting the complex pathophysiology of postoperative ileus and the influence of variables such as surgical technique, perioperative care pathways, analgesic regimens, and patient selection.

Future research should prioritize the standardization of PPOI definitions and outcome measures to improve comparability across studies, incorporating consistent criteria for symptomatology and postoperative timing and adopting universally accepted units of measurement. Well-designed trials specifically targeting perioperative risk stratification and predictive modeling are needed, as current evidence is largely derived from intervention-focused studies. In addition, further investigation into biological markers and mechanistic pathways may help identify patients at high risk and enable more personalized perioperative management strategies.

## 5. Conclusions

Prolonged postoperative ileus remains a complex and clinically relevant complication after colorectal surgery, occurring within the interaction of multiple perioperative factors rather than a single determinant. Individual randomized intervention–control comparisons identified potentially favorable effects of several multimodal analgesic, pharmacological, and non-pharmacological perioperative strategies on PPOI and/or gastrointestinal recovery; however, the magnitude and consistency of these effects varied across trials and endpoints. Importantly, 7 of the 36 included RCTs (19.4%) reported an operational definition of POI/PPOI, but only 5 of these (13.9% of all included trials) applied a predefined time-based definition corresponding specifically to PPOI. Consequently, most of the available evidence was based on less clearly defined POI/PPOI outcomes or surrogate measures of gastrointestinal recovery.

Heterogeneity in PPOI definitions, interventions, perioperative pathways and outcome measures, together with methodological limitations in several trials, limits definitive conclusions regarding the efficacy of individual strategies for PPOI prevention. Moreover, associations involving factors that were not independently randomized, including ERAS adherence, bowel preparation, opioid exposure, postoperative inflammation, and surgical approach in most trials, should not be interpreted as independently causal. Nevertheless, the findings identify several potentially promising perioperative strategies associated with improved postoperative gastrointestinal recovery, although their efficacy in preventing clinically defined PPOI remains uncertain.

Future research should focus on harmonizing PPOI definitions and identifying robust, clinically applicable predictors to enable more targeted prevention and optimization of perioperative care, with greater emphasis on readily available biochemical and inflammatory laboratory markers that can be assessed in routine practice across healthcare settings, enabling broad applicability and early identification of patients at risk.

## Figures and Tables

**Table 1:** Population characteristics.

Author, Year	Total Number of Patients (n)	Patients in Intervention Group (n)	Intervention	Age (Mean ± SD or Median/Range)	Sex (M:F)	Pathology	Previous Abdominal Surgery (n/%)	ASA Score II (n)/III (n)	LOS (Mean ± SD or Median/Range) (Days)
^ Quah et al., 2006 [47]	38	19	Gum chewing	67 ± 9.7	13:6	Malignant	5/26%	NA	9.4 ± 2.5; NA; NA
* MacFater et al., 2022 [54]	56	28	Intraperitoneally local anesthetic	74.5/(57–89)	15:11	Malignant/Benign	NA	16/9	5/(3–88); NA; NA
	56	28	Intravenous local anesthetic	68/(45–84)	14:14	Malignant/Benign	NA	19/6	5/(3–11)
*° Peters et al., 2018 [43]	265	132	Lipid-enriched enteral nutrition	69/(62–75)	80:52	Malignant	49/37%	96/15	6.0/(4.0–10.0)
# Nasseri et al., 2023 [37]	99	31	Warm water	61.6 ± 15.5	20:11	Malignant/Benign		20/11	4.0/(3.0–5.0)
	99	31	Decaffeinated coffee	63.7 ± 14.7	19:12			16/15	3.0/(3.0–4.0)
	99	37	Caffeinated coffee	59.5 ± 15.0	22:15			23/14	4.0/(3.0–6.0)
^ Ostovic et al., 2025 [41]	82	42	Lidocaine/Ketamine +Placebo	L:62 ± 7/K:63 ± 7	L: 23:19/K: 24:18	Malignant	NA	L:33; K:29/L:7;K11	NA
	82	21	Lidocaine +Ketamine	NA				NA	NA
# Sim et al., 2007 [34]	79	40	COX-2-specific inhibitor	61.0 ± 11.4	20:20	Malignant/Benign	NA	NA	4.9 ± 2.7
*° Paterson et al., 2025 [36]	557	278	Intravenous Lidocaine	65.9 ± 11.0	157:122	Malignant/Benign	NA	NA	6.0 ± 5.9;
# Ludwig et al., 2008 [50]	654	329	Alvimopan	59.9 ± 14.4	165:164	Malignant/Benign	Exclusion criteria	NA	NA
*° Milne et al., 2022 [48]	148	74	Prucalopride	71/(64–78)	45:29	Malignant/Benign	NA	43/23	4/(2–6)
# Silinsky et al., 2021 [30]	55	28	Meloxicam	58.8 ± 11.2	15:12	Malignant/Benign	NA	NA	3.59 ± 1.77
^ Martellucci et al., 2021 [39]	340	170 (AS85:SS85)	Right colon and active Tibial nerve stimulation	71/(43–89)	90:80	Malignant	NA	96/45	5/(3–11)
	340	170 (AS85:SS85)	Left Colon and active Tibial nerve stimulation	67/(37–92)	100:70			81/52	4/(3–16)
^ Matros et al., 2006 [59]	66	22	Chewing gum	62 ± 14	8:14	Malignant	NA	15/6	119/(99–138)
^ Parnasa et al., 2021 [42]	58	30	Coffee	56.90 ± 12.77	15:15	Malignant/benign	10/33.3%	17/2	6.0/(5–8)
^ Schlachta et al., 2007 [35]	44	22	Intravenous Ketorolac	59.5 ± 8.2	12:10	Malignant/benign	NA	NA	4.0
^ Hayashi et al., 2019 [32]	46	23	Coffee	74.0/(48.0–90.0)	5:18	Benign	12/52.2%	18/3	NA
*° Yang et al., 2018 [38]	89	46	Chewing Xylitol Gum	62.70 ± 7.84	31:12	Malignant	Exclusion criteria	NA	NA
^ Schuster et al., 2006 [49]	34	17	Chewing sugarless gum	60 ± 6.1	11:6	Recurrent diverticulitis/Malignant	3/17%	NA	4.30 ± 0.43
^ Smith et al., 2000 [29]	134	65	Erythromycin	63.2 ± 12.6	43:22	Malignant	0/0%	NA	NA
^ Swenson et al., 2010 [40]	42	20	Epidural bupivacaine	49/(36–54)	16:4	Malignant/Benign	NA	18/0	NA
	42	22	Intravenous lidocaine	52/(40–62)	10:12			14/7	NA
# Taqi et al., 2007 [46]	50	25	Thoracic epidural analgesia	65 ± 16.18	14:11	Malignant/Benign	NA	17/5	5/(4.65–6.16)
# Kitano et al., 2017 [45]	1057	528	Open D3 dissection	64/(57–69)	312:215	Malignant	Appendectomy exception	NA	NA
	1057	529	laparoscopic D3 dissection	64/(28–69)	282:247				
^ Forrester et al., 2014 [63]	47	34	Chewing sugar-free gum	60.13/(31–94)	14:33	Recurrent diverticulitis/Malignant	Exclusion criteria	NA	6.31
^ Dulskas et al., 2015 [53]	90	30	Decaffeinated coffee	62.4 ± 10.8	16:14	Malignant	NA	NA	6.6
	90	30	Caffeinated coffee	67.3 ± 6.8	16:14		NA	NA	6.0
# Lee et al., 2013 [52]	98	46	Early rehabilitation program	61.2 ± 10.8	36:16	Malignant	Exclusion criteria	22/4	7.5/(7–11)
# Ahn et al., 2013 [55]	31	17	Postoperative inpatient exercise program	55.61 ± 7.11	12:5	Malignant	NA	NA	7.82 ±1.07
# Da Fonseca et al., 2011 [58]	50	26	Early oral feeding	57.4 ± 16.3	8:16	Malignant/Benig	NA	10/1	4.0 ± 3.7
^ Herzog et al., 2011 [57]	107	50	Intravenous choline citrate	63.8 ± 12.1	24:26	Malignant/Benign	NA	NA	10.4 ± 2.1
# Gervaz et al., 2010 [44]	113	59	Laparoscopic sigmoid colectomy	57/(29–82)	30:29	Diverticulitis	30/50%	45/4	5/(4–69)
	113	54	Conventional open sigmoid colectomy	62.5/(38–84)	23:31		31/57%	39/2	7/(5–17)
^ Zingg et al., 2008 [31]	169	83	Oral bisacodyl	67.9 ± 13.2	42:41	Malignant/Benign	NA	NA	16 ± 12
# Haase et al., 2005 [56]	60	20	Guided imagery	64.7 ± 8.6	11:9	Malignant	NA	NA	NA
	60	22	Muscle relaxation	64.8 ± 9.9	14:8				
# Espin-Basany et al., 2005 [62]	300	100	Three doses of oral antibiotic	66.6	63:37	Malignant/Benign	NA	NA	10.8/(4–67)
	300	100	One dose of oral antibiotic	67	67:33				
	300	100	No oral antibiotics	69	62:38				
* Colibaseanu et al., 2019 [51]	200	98	Intrathecal opioid	58/(49–69)	55:43	Malignant/Benign	NA	NA	3 ± 3, 4
	200	102	Transversus abdominis plane block	61/(50–68)	57:45	Malignant/Benign	NA	NA	3 ± 3, 3
^ Chen et al., 2005 [64]	74	35	Morphine	68/(47.8—74.0)	22:13	NA	NA	NA	15/(14.0–19.3)
	74	39	Morphine + ketorolac	64.5/(48.5—71.0)	19:20				14.5/(12.0–20.0)
^ Chen et al., 2009 [61]	102	50	Morphine	60.5 ± 12.2	30:20	NA	NA	NA	NA
	102	52	Morphine + ketorolac	57.3 ± 11.6	23:29				
^ Wiriyakosol et al., 2007 [33]	20	10	Bisacodyl suppository	63.8 ± 8.1	5:5	Malignant	NA	8/2	8.5 ± 2.7
*° Law et al., 2002 [60]	80	42	Loop ileostomy	65.2	26:16	Malignant	NA	NA	9
	80	38	Loop transverse colostomy	67.8	23:16				9

N—number; M—masculine; F—feminine; LOS—length of stay; ASA—American Society of Anesthesiologists Physical Status Classification; AS—active stimulation; SS—sham stimulation; NA—not applicable; SD—standard deviation; * Operational definition POI/PPOI (n = 7); *° Predefined time-based operational definition corresponding specifically to PPOI (n = 5, subset of the seven studies reporting an operational POI/PPOI definition); # POI/PPOI reported without a sufficiently detailed predefined PPOI definition (n = 12); ^ Gastrointestinal recovery assessed primarily through surrogate outcomes, without a predefined PPOI definition (n = 17).

**Table 2:** Intraoperative characteristics.

	Stoma (Total n. Studies/n. Stoma)	Operation Duration (min.) Mean ± sd or Median [Range] (Min/Max)	IO Bloodloss mL Mean ± sd or Median [Range] Min/Max; Transfusion n. Patients/%
Classic	13/3	108 ± 39/271.6 ± 74.8	111 ± 85/260 ± 222; NA
Laparoscopic	10/1	102.0 ± 37.2/231.2 ± 53.8	18.4 (0.0–260.0)/177.1 ± 107.0; 2/8%
Mixed (C/L/R/co)	14/3	145 (119–185)/244.2 ± 26.4	30 (20.0–50.0)/521; NA

n—number, (min.)—minutes; IO—intraoperative; sd—standard deviation, min—minimum, max—maximum, C—classic, L—laparoscopy, R—robotic, co—conversion.

**Table 3:** Postoperative outcomes according to intended analgesic strategy.

Intended Analgesia	Need for NGT Reinsertion (%) Patients	Readmission (n) Days	Reoperation (n) Patients	First Flatus (Days) Range	First Bowel Movement (Days) Range
Opioid/Multimodal opioid	3.3–18 *	0–1 *	0–3 *	1.1–4.1	0.8–5.2
Non-opioid/Multimodal Opioid-sparing	0–13.5 *	1–4	0–1	1.5–2.0	2.0–3.0
Regional + Opioid	0	0	1–4	1.0–2.7	2.0–3.0
Regional + Non-opioid	NR	0–2	NR	0.3–2.4	2.2–3.2

* Only studies with available numeric data were included; several studies in this category did not report NGT reinsertion, readmission, or reoperation; n—number; NR—not reported; NGT—nasogastric tube; %—percent.

**Table 5:** Risk-of-bias assessment of the included randomized controlled trials using the Cochrane Risk of Bias 2 (RoB 2) tool.

Domain	Low Risk	Some Concerns	High Risk
D1	52.8%	47.2%	0%
D2	33.3%	44.4%	22.2%
D3	47.2%	50.0%	2.8%
D4	58.3%	41.7%	0%
D5	47.2%	47.2%	5.6%
Overall	27.8%	44.4%	27.8%

D1—bias arising from the randomization process; D2—bias due to deviations from intended interventions; D3—bias due to missing outcome data; D4—bias in measurement of the outcome; D5—bias in selection of the reported result; % represent the proportion of the 36 included randomized controlled trials assigned to each risk-of-bias category for the respective domain and overall assessment.

**Table 6:** GRADEpro certainty of events.

Certainty Assessment							No. of Patients	Certainty
No. of Studies	Study Design	Risk of Bias	Inconsistency	Indirectness	Imprecision	Other Considerations		
PPOI incidence								
10	randomised trials	not serious	very serious	not serious	not serious	none	1532/2894 (52.9%)	⨁⨁◯◯ Low
	First Flatus							
28	randomised trials	serious	serious	not serious	serious	none	1247/2530 (49.3%)	⨁◯◯◯ Very low
First Bowel Movement								
27	randomised trials	serious	serious	not serious	serious	none	1235/2318 (53.3%)	⨁◯◯◯ Very low
Nasogastric tube reinsertion								
11	randomised trials	not serious	not serious	not serious	serious	none	1155/2343 (49.3%)	⨁⨁⨁◯ Moderate
Length of Hospital Stay								
26	randomised trials	serious	not serious	not serious	not serious	none	1656/3078 (53.8%)	⨁⨁⨁◯ Moderate

## Data Availability

The original contributions presented in this study are included in the article/Supplementary Materials. Further inquiries can be directed to the corresponding authors.

## Supplementary Materials

The following supporting information can be downloaded at: https://www.mdpi.com/article/10.3390/jcm15218532/s1, Table S1. PRISMA Checklist; Table S2. Search key strategy.
